# Nanomedicine Approaches for Intervertebral Disc Regeneration: From Bench to Bedside

**DOI:** 10.3390/pharmaceutics17030313

**Published:** 2025-02-28

**Authors:** Yifan Ding, Fan Li, Yunyun Wang, Weizhen Pan, Xiangning Fu, Songwei Tan

**Affiliations:** 1 Department of Thoracic Surgery, Tongji Hospital, Tongji Medical College, Huazhong University of Science and Technology, Wuhan 430030, China; dingyifan@tjh.tjmu.edu.cn (Y.D.); tjhtsdrli@tjh.tjmu.edu.cn (F.L.); 2 Department of Cardiology, the Fifth Hospital of Wuhan, Jianghan University, Wuhan 430030, China; m201875994@alumni.hust.edu.cn; 3 School of Pharmacy, Tongji Medical College, Huazhong University of Science and Technology, Wuhan 430030, China; panweizhen@hust.edu.cn

**Keywords:** intervertebral disc degeneration, nanomedicine, drug delivery, inflammation, regenerative medicine

## Abstract

Intervertebral disc degeneration (IDD) is a leading cause of low back pain (LBP) and neurological dysfunction, contributing significantly to disability-adjusted life years globally. The progression of IDD is driven by excessive oxidative stress, inflammation, apoptosis, and fibrosis, which disrupt the balance between anabolic and catabolic processes, leading to extracellular matrix (ECM) degradation and IDD. Current treatment options, such as conservative therapy and surgical intervention, are limited in halting the disease progression and often exacerbate degeneration in adjacent discs. This review highlights the challenges in treating IDD, particularly due to the limited drug delivery efficiency to the intervertebral disc (IVD). It explores the potential of nanobiomedicine and various nanomaterial-based delivery systems, including nanoparticles, microspheres, gene-nanocomplexes, fullerene, exosomes, and nanomaterial-composite hydrogels. These advanced delivery systems can enhance targeted drug delivery, improve local drug concentration, and sustain drug retention within the IVD, offering promising therapeutic strategies to address IDD. The review also examines the therapeutic effects of these nanomaterials on IDD, focusing on their impact on metabolism, inflammation, apoptosis, fibrosis, and stem cell migration and differentiation, aiming to provide innovative strategies for intervertebral disc regeneration.

## 1. Introduction

Intervertebral disc degeneration (IDD)-related diseases are major contributors to low back pain and neurological dysfunction. The disability-adjusted life years lost due to IDD are significantly higher than those of other diseases, making it a leading cause of disability and imposing a substantial economic burden worldwide [1]. The etiology of LBP is highly complex, involving various factors, with lumbar IDD being the primary cause [2]. IDD is a complex process characterized by the imbalance between anabolic and catabolic processes within the intervertebral disc (IVD), ultimately leading to alterations in the extracellular matrix (ECM). This imbalance results in a reduction in the number of nucleus pulposus cells (NPCs), excessive oxidative stress, and inflammation. Among these, inflammation, apoptosis, and fibrosis play pivotal roles in the progression of degeneration [3,4]. Inflammatory responses accelerate catabolic processes, while disc cell apoptosis diminishes the anabolic capacity, and fibrosis disrupts the disc microenvironment. These changes collectively exacerbate the metabolic imbalance within the IVD.

Common treatments for IDD-related diseases include conservative therapy and surgical intervention. Conservative therapy primarily involves the use of nonsteroidal anti-inflammatory drugs and physical therapy, which can alleviate pain to some extent but cannot halt the progression of IDD. Surgical treatment effectively relieves pain but may exacerbate degeneration in adjacent discs. Therefore, there is an urgent need to develop new therapeutic approaches for IDD.

As the largest avascular tissue in the human body [5], the IVD faces limitations in traditional drug treatments due to wide drug distribution and rapid metabolism, which result in low drug concentrations and short residence times. Therefore, targeted drug delivery to the IVD is crucial to enhance local drug concentration and retention time. Nanobiomedicine has become an important branch of nanotechnology. The size and physicochemical properties of nanoparticles determine their ability to deliver chemical drugs and various biological molecules. Nanomaterials possess characteristics such as small size, large specific surface area, ease of surface modification, high drug loading capacity, and controlled drug release [6], making them highly promising for applications in intervertebral disc regeneration and repair.

This review categorizes and discusses common nanomedicine delivery systems for IDD, including nanoparticles, microspheres, gene-nanocomplexes, fullerene, exosomes, and nanomaterial-composite hydrogels. It explores their applications in targeted drug delivery to the IVD and evaluates their therapeutic effects on IDD through mechanisms such as metabolism, inflammation, apoptosis, fibrosis, and the promotion of stem cell migration and differentiation. The aim is to provide new nanomedicine-based therapeutic strategies for intervertebral disc regeneration.

## 2. Mechanisms and Therapeutic Approaches for IDD

**Figure 1:**
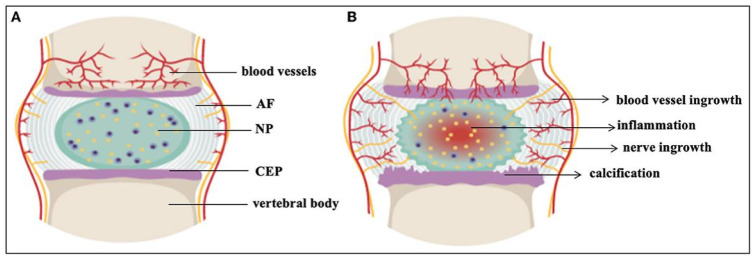
The IVD. (**A**) Healthy IVD. (**B**) Degenerated IVD. AF, annulus fibrosus; CEP, cartilaginous end plates; NP, nucleus pulposus; the black dots: notochord cells; the yellow dots: notochord-like cells. Reproduced with permission [9]. Copyright 2023 Samanta, Lufkin and Kraus.

**Figure 2:**
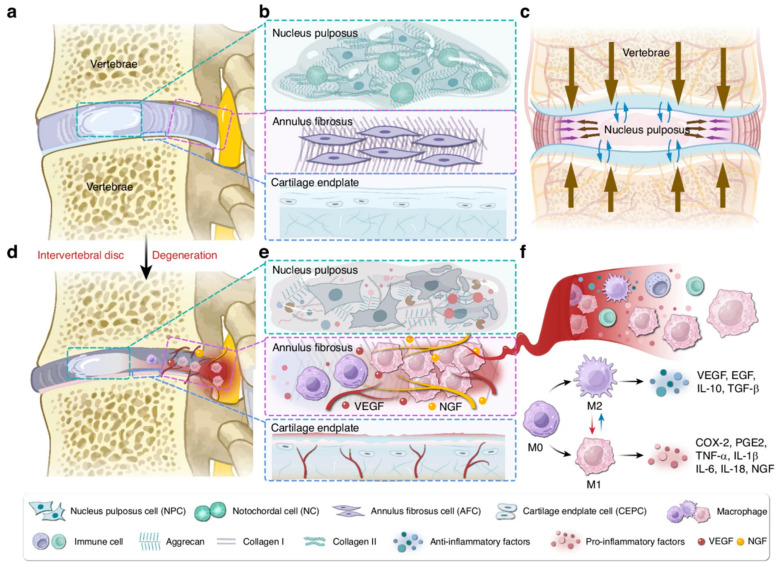
A schematic representation of the healthy IVD, degenerated IVD, and the inflammatory pathological microenvironment. (**a**) Overview of the IVD. (**b**) Cellular composition and ECM in the healthy IVD. (**c**) Mechanical function of the IVD. (**d**) IDD and herniation. (**e**) Cellular and ECM alterations in degenerated IVD. (**f**) Macrophage infiltration and polarization. Reproduced with permission [18]. Copyright 2025 Springer Nature.

As shown in Figure 1A and Figure 2a,b, the IVD consists of the nucleus pulposus (NP), annulus fibrosus (AF), and cartilage endplate (CEP). The NP contains a substantial amount of ECM and a small population of nucleus pulposus cells (NPCs). The NPCs comprises notochordal cells and notochordal-like cells. During embryonic development, notochordal cells are generated through a series of meticulously orchestrated events. With advancing age, these notochordal cells undergo transdifferentiation into notochordal-like cells. The main components of NP are proteoglycans (Aggrecan, Acan) and type II collagen (Collagen 2a1, Col2a1). Surrounding the NP is the AF, with the outer layer consisting of fibroblast-like cells and the inner layer consisting of round chondrocyte-like cells. From the NP to the outer layer of the AF, Col2a1 gradually decreases, while type I collagen (Collagen 1, Col1) increases. Together, the NP and AF form a system with a certain hydrostatic pressure, capable of tolerating tensile forces, bearing pressure, and distributing load (Figure 2c), thereby coordinating spinal movement [7]. The CEP is the only medium for blood supply between the vertebral body and the IVD. When the disc is compressed, water is exuded through the CEP, and when the pressure is relieved, it re-enters through the CEP, nourishing the IVD [8].

As shown in Figure 1B and Figure 2d–e, during IDD, the boundaries between the NP and AF become gradually blurred, and the content of proteoglycans and Col2a1 decreases. The hydration ability and osmotic pressure also decline. As degeneration progresses, Col1 gradually increases, while Col2a1 decreases. The biomechanical properties of the disc change, and alterations in factors such as compressive load, shear stress, and elastic modulus exacerbate the degeneration of the disc [10]. Subsequently, partial or complete rupture of the AF occurs, causing the NP and/or CEP to protrude outward, compressing the sinuvertebral nerve and nerve roots, resulting in lumbar and leg pain [11]. Additionally, exposed NP can induce macrophage infiltration, releasing a large amount of inflammatory mediators such as IL-1β and TNF-α, promoting angiogenesis and nerve ingrowth, increasing the excitability of nociceptive neurons. (Figure 2f) These inflammatory factors further upregulate the activity and expression of matrix metalloproteinases (MMPs), leading to the degradation of Col2a1 and proteoglycans, further aggravating IDD [12,13].

Common therapeutic approaches for IDD-related diseases include conservative and surgical treatments. Conservative treatment primarily involves the use of non-steroidal anti-inflammatory drugs, physical therapy, and other modalities, which can alleviate pain to some extent but cannot halt the progression of IDD. Surgical options include simple discectomy, spinal fusion, and artificial disc replacement. In patients with typical clinical symptoms and imaging findings, surgical intervention often yields satisfactory outcomes, with spinal fusion being the most commonly applied technique [14]. However, surgery is highly invasive and may accelerate degeneration of adjacent segments. Therefore, there is an urgent need to find new methods to address the challenges posed by IDD. The development of nanomedicine offers new strategies for the treatment of IDD, particularly with significant advantages in enhancing drug bioavailability, reducing side effects, and improving therapeutic targeting.

Firstly, micro/nanocarriers can achieve targeted delivery through physical, chemical, or biological interactions, ensuring that drugs are accurately delivered to the affected area of the degenerated IVD. Studies have shown that using micro/nanoparticles as carriers allows drugs to be stably carried in the bloodstream and precisely released to the target tissue, reducing the side effects of the drugs on non-target tissues. Moreover, the tunability of nanocarriers allows them to respond to specific environmental stimuli (such as pH, temperature, or ROS levels) during drug release, enabling more precise control. For example, Ma et al. [15] designed a microsphere of hyaluronic acid methyl methacrylate containing Schiff base bonds for targeted delivery to degenerated IVD and pH-responsive drug release. After loading with bovine serum albumin nanoparticles, the system enhanced the sustained-release performance of IL-1Ra, aiming to reverse the inflammatory microenvironment. We synthesized ROS-responsive polymer nanoparticles, exhibiting excellent stability in vitro [16]. It significantly enhanced local sustained release and achieved targeted delivery of luteolin to the IVD, demonstrating effective anti-inflammatory properties.

Therefore, nanomaterials have shown great potential in IDD, enabling targeted drug delivery, prolonged circulation, and excellent biocompatibility and availability, providing new pathways for the treatment of IDD [17].

## 3. Nanomedicine Provides New Perspectives for the Treatment of IDD

Nanomedicine has emerged as a promising strategy for the treatment of IDD, offering new ways to overcome the limitations of conventional therapies. By utilizing nanomaterials, targeted drug delivery and multifunctional smart adjustable nanoparticles are now achievable, improving treatment precision and minimizing side effects.

Drug delivery systems for IDD must satisfy several critical requirements to ensure therapeutic efficacy and safety. These include biocompatibility, targeted delivery capability, stability in the harsh disc microenvironment, controlled release properties, and the ability to penetrate the dense extracellular matrix of the intervertebral disc [19,20]. Not all nanoparticles are suitable for intervertebral therapy, as their design must be tailored to address the unique challenges of the disc environment, such as low cellularity, limited nutrient supply, and high mechanical load [21,22]. Recent advances in nanotechnology have enabled the development of functionalized nanoparticles that meet these requirements, offering promising potential for targeted drug delivery in IDD. Yang et al. [23] designed an engineered macrophage membrane-coated MnO2 nanoparticle that targets and adsorbs nerve growth factor within the IVD, modulates macrophage polarization, and inhibits the local overproduction of ROS. However, further research is needed to optimize these systems for clinical translation.

Recent published reviews have primarily focused on local injection therapies, outlining the use of common nanoparticles, polymeric nanoparticles, and nanocomposite hydrogels for IDD treatment [24,25]. In this review, we place greater emphasis on targeted delivery to the IVD based on different administration routes and elucidate the role of nanomaterials in promoting IVD regeneration from perspectives such as enhancing stem cell migration and differentiation, anti-inflammatory effects, and anti-fibrotic actions. This review discusses the development and applications of nanoparticles in a structured manner, beginning with non-biological source nanoparticles categorized by size, including nanoparticles, gene-nanoparticle complexes, fullerene derivatives (fullerol), and microspheres. It then transitions to biological source nanocarriers, focusing on exosomes, and concludes with an overview of nanocomposite hydrogels as advanced functional materials.

### 3.1. Drug Delivery Routes for IDD Therapy

In current research on IDD, commonly employed drug delivery routes include local injection, intraperitoneal injection, and intravenous injection. Local injection is the most commonly employed drug delivery approach, as it allows for direct targeting of the affected area. However, local injection is invasive and may cause tissue damage and side effects, limiting its widespread application. A study by Elmounedi et al. [26] found that a 29G needle did not induce disc degeneration, whereas a larger 21G needle caused additional damage. Research by Mao et al. [27] demonstrated that an injection volume of less than 2 µL did not lead to IDD, whereas doses exceeding 2 µL resulted in significant IDD. In clinical translation studies involving humans, local injections must penetrate thick subcutaneous tissue, muscle, ligaments, and other relatively rigid soft tissues, making accurate control of the injection dosage and needle size challenging. This poses significant challenges for the selection of needles and injection dosages in clinical research. Furthermore, in our study on liposomal nanoparticles, intraperitoneal injection was employed and IVD targeting was achieved [28]. While intraperitoneal injection is widely used in animal experiments, it is rarely applied in human clinical studies. Intravenous administration is extensively utilized in both basic research and clinical trials; however, given that the IVD is the largest avascular tissue in the human body, this route may result in inadequate drug delivery to the disc. Oral administration, being the safest and most compliant method, remains unexplored in basic research on IDD. Encouragingly, recent studies have shown that yeast microcapsules as nanodrug carriers exhibit promising efficacy in osteoarthritis [29,30], enabling targeted delivery to arthritic sites via the oral route. This novel oral targeted delivery approach may also be applicable to IDD. Our research team is currently investigating this area, and we believe that relevant results will address the gap in research on oral targeted drug delivery for IVD regeneration.

### 3.2. Nanoparticles

#### 3.2.1. IVD Targeted Delivery

Due to the avascular nature of the IVD, conventional drug treatments often fail to penetrate effectively, limiting their therapeutic efficacy [31]. Nanotechnology offers a promising solution by enabling targeted drug delivery through surface-modified nanoparticles, reducing systemic dosage and minimizing side effects [32]. Targeted formulations can be classified into passive, active, and physicochemical targeting systems.

Passive targeting relies on the natural accumulation of nanoparticles in regions rich in mononuclear–macrophage cells. Our research group [28] synthesized liposomal nanoparticles loaded with oxymatrine, which exhibit good sustained-release properties. Due to the passive targeting ability of the liposomes, this system can localize in degenerated IVD, increasing drug accumulation and reducing side effects. Moreover, oxymatrine encapsulated in the nanoparticles can suppress inflammation in the IVD, promote ECM synthesis, and facilitate disc repair and regeneration. This passive targeting may be attributed to the accumulation of macrophages in the degenerated disc region, which facilitates the IVD target of the liposomes.

Active targeting involves modifying nanoparticles to direct drugs to specific regions. The research group of Xudong Li [33] developed a fullerene nanoplatform capable of targeting macrophages. By conjugating carboxylated fullerenes with the primary amine group of a specific peptide that binds to the formyl peptide receptor-1 on macrophage surfaces, they synthesized the composite nanoparticles. Electron paramagnetic resonance spectroscopy revealed that this system effectively scavenges free radicals (hydroxyl and superoxide anions) and specifically binds to macrophages, reducing the expression of IL-6, IL-1, TNF-α, and COX-2. In vivo studies involving tail vein injection in C57/B6L mice confirmed the excellent targeting capability of this system to the IVD in an acupuncture-induced degeneration model, alleviating sciatic nerve pain symptoms in mice 12 days post-surgery. This study demonstrates active macrophage-targeted delivery via peptide-modified nanofullerene carriers, offering a novel strategy for the biological therapy of IDD.

Physicochemical targeting refers to the application of physical and chemical methods to achieve targeted drug delivery. The IVD is avascular, making it difficult for drugs to penetrate; however, its proximity to the adjacent vertebrae and superficial location of the spine create an opportunity for the application of physicochemical factors in vivo to achieve targeting delivery. Among these, ultrasound stimulation is a simple and feasible approach. Nguyen et al. [34] encapsulated simvastatin in nano-microdroplets, and exposure of this system to high-intensity focused ultrasound triggered the disintegration of the microdroplets, leading to the release of simvastatin. The nanocarrier system remained stably present in rabbit IVD tissue for at least 14 days. When cultured with NPCs in medium containing nano-droplets for 24 h, the cell viability remained above 85%, demonstrating excellent biocompatibility.

Furthermore, researchers have innovatively proposed combining physicochemical targeting with active targeting for the treatment of IDD, demonstrating promising clinical application prospects. Shen et al. [35] developed a novel ultrasound-mediated poly(lactic acid)-poly(glycolic acid) copolymer nanobubble (NBS) delivery system to enhance the targeting efficacy for nucleus pulposus cells in the treatment of intervertebral disc degeneration. First, the NBS loaded with resveratrol (RES) was synthesized using the re-emulsification method. The targeting antibody for the nucleus pulposus biomarker CDH2 (AbCDH2) was conjugated to the NBS via carbodiimide coupling. The physical properties, cell-specific targeting ability, and in vitro and in vivo anti-metabolic effects of the RES/AbCDH2-NBS were subsequently evaluated. RES/AbCDH2-NBS can trigger rapid release of RES upon ultrasound exposure. In a rabbit IDD model, ultrasound-mediated in situ injection of RES/AbCDH2-NBS effectively delayed IDD in vivo. This study revealed the potential clinical therapeutic value of RES/AbCDH2-NBS for IDD through the dual targeting approach of ultrasound and nucleus pulposus cell biomarker antibodies.

#### 3.2.2. Promotion of Anabolic Metabolism

IDD is accompanied by an imbalance between catabolic and anabolic processes, characterized by a decline in anabolic capacity and a dominance of catabolic activity. Balancing these two processes is crucial for the repair and regeneration of the NP tissue [10]. It has been reported that certain growth factors and bioactive peptides can effectively promote anabolic metabolism in the NP, restoring its normal phenotype [23]. Moreover, due to their ability to enhance cell biocompatibility and hydrophilicity, nanomaterials exhibit significant potential for facilitating ECM generation within the IVD.

**Figure 3:**
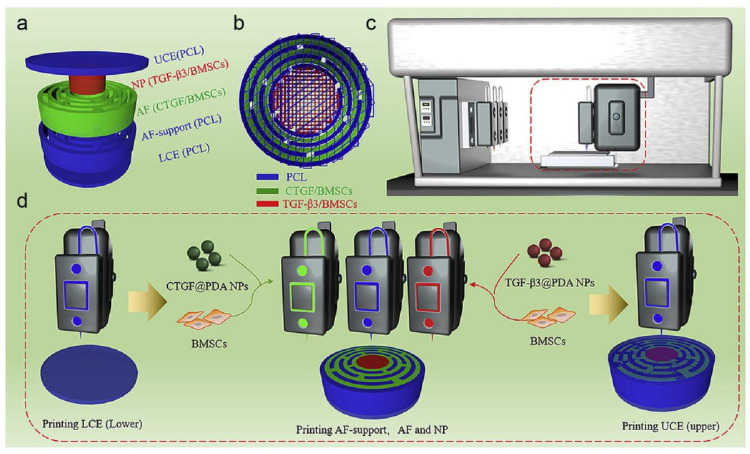
Development of a dual-growth factor (GF)-releasing IVD scaffold using 3D bioprinting technology. (**a**) Three-dimensional model of the IVD scaffold. (**b**) Printing trajectory for the IVD scaffold using 3D bioprinting. (**c**) 3D bioprinting equipment. (**d**) Printing process for fabricating the IVD scaffold via 3D bioprinting. Reproduced with permission [36]. Copyright 2021, Elsevier.

As shown in Figure 3a,b, Sun et al. [36] employed 3D bioprinting technology to load connective tissue growth factor (cTGF) and transforming growth factor-β3 (TGF-β3) into polydopamine nanoparticles, forming a dual-growth factor release-based IVD scaffold. In Figure 3c,d, a dual-factor releasing IVD scaffold is implanted with BMSCs to form a nanomaterial-loaded 3D bioprinted composite delivery system. This composite system was able to induce MSCs differentiation into nucleus pulposus-like cells and annulus fibrosus-like cells. Upon subcutaneous implantation in nude mice, immunohistochemical analysis revealed that the scaffold mainly consisted of Col2a1. This nanostructured dual-growth factor release scaffold demonstrated excellent biological activity and the ability to promote ECM generation. Furthermore, a bioactive peptide—poly(γ-glutamic acid) (γ-PGA)—was used for surface modification of the scaffold, which was shown to support rat chondrocyte culture and promote chondrocyte growth and differentiation [37]. In this study, a poly(γ-glutamic acid) injection was patented for the treatment of degenerative joint diseases [10]. Subsequently, Antunes et al. [38] designed a poly(γ-glutamic acid)/chitosan nanocomposite, which facilitated the synthesis of glycosaminoglycans and Col2a1 in NPCs. Chitosan nanoparticles helped γ-PGA fully interact with degenerated IVD tissue and delayed its action time. In an in vitro organ culture model of bovine intervertebral discs, poly(γ-glutamic acid)/chitosan nanocomposite promoted the regeneration of degenerated bovine discs, restoring early ECM loss and maintaining annulus fibrosus integrity.

#### 3.2.3. Promotion of Stem Cell Migration and Differentiation

With the gradual understanding of the mechanisms underlying IDD and the advancement of stem cell technologies, stem cell transplantation for IVD repair and the restoration of normal spinal physiology have gained increasing attention from both scientists and clinicians [39]. Stem cells possess strong self-proliferation, multilineage differentiation potential, and immune tolerance. Under specific conditions, they can be induced to differentiate into nucleus pulposus-like chondrocytes, expressing Col2a1 and proteoglycans, similar to the ECM secreted by NPCs, thereby contributing to disc repair and immune modulation. In 2003, researcher Sakai was the first to apply stem cells in IDD therapy [40], and the results demonstrated that stem cell transplantation could inhibit IDD. Zhang et al. [41] synthesized albumin/heparin nanoparticles (BHNPs) with a particle size of approximately 110 nm as a delivery vehicle for stromal cell-derived factor-1a (SDF-1a, also known as C-X-C motif chemokine 12), to protect the factor from degradation and achieve controlled release. The nanoparticles exhibited high SDF-1a loading capacity and sustained release characteristics. In the in vitro Transwell model, BHNPs/SDF showed satisfactory effects in inducing bone marrow-derived mesenchymal stem cell (MSCs) migration, promoting MSC migration to degenerated IVD, and facilitating IVD repair and regeneration. Compared to SDF-1a and BHNPs, BHNPs/SDF exhibited significantly better regenerative effects on damaged IVD. Gan et al. [42] employed the dextran/gelatin gel-encapsulated MSCs and transforming growth factor-β3 (TGF-β3) complex nanoparticle co-delivery system to induce MSCs to differentiate into nucleus pulposus-like cells. After intervention with this nanocarrier system, the expression of NP cell phenotype markers, KRT18 and Sonic hedgehog, was significantly increased in MSCs, indicating that the co-delivery system effectively induced directed differentiation into MSCs in situ. Additionally, the aldehyde groups of the composite gel can react with the surface of biological tissues, causing the hydrogel to covalently bind to the tissue surface, preventing leakage of the encapsulated MSCs and TGF-β3. This lays the foundation for stem cell transplantation in the repair and regeneration of NP tissue.

### 3.3. Gene-Nanoparticle Complexes

Gene therapy refers to the process of introducing specific genes into target cells to exert therapeutic effects, thereby treating diseases. The key challenge in gene therapy is how to deliver the target gene into the target cells. Viral vectors have the advantage of high transfection efficiency, but their biological safety concerns limit their application [43]. Studies have shown that gene therapy mediated by viral vectors can cause paralysis of the lower limbs in experimental animals [44]. Recently, with the development of nanotechnology, there has been an increasing amount of research on the use of nanomaterials for nucleic acid delivery, demonstrating their potential application capabilities [45]. Gene delivery using nanomaterials has several advantages: as a type of non-viral vector, nanomaterials exhibit good biocompatibility and biodegradability, do not induce immunogenicity, enhance cell uptake through endocytosis, and improve transfection efficiency. Their controlled release effect prolongs the duration of gene expression, and surface modifications enable targeted delivery [46]. Non-viral vectors include cationic liposomes, cationic polymers, and dendritic macromolecules, which electrostatically bind with DNA to form gene-nanoparticle complexes with sizes ranging from tens to hundreds of nanometers. This prevents gene degradation and facilitates its uptake, thereby exerting specific biological effects. The following review will focus on nanoparticle-mediated anti-inflammatory, anti-fibrotic, and stem cell migration and differentiation applications, as shown in Table 1.

#### 3.3.1. Anti-Inflammation

**Figure 4:**
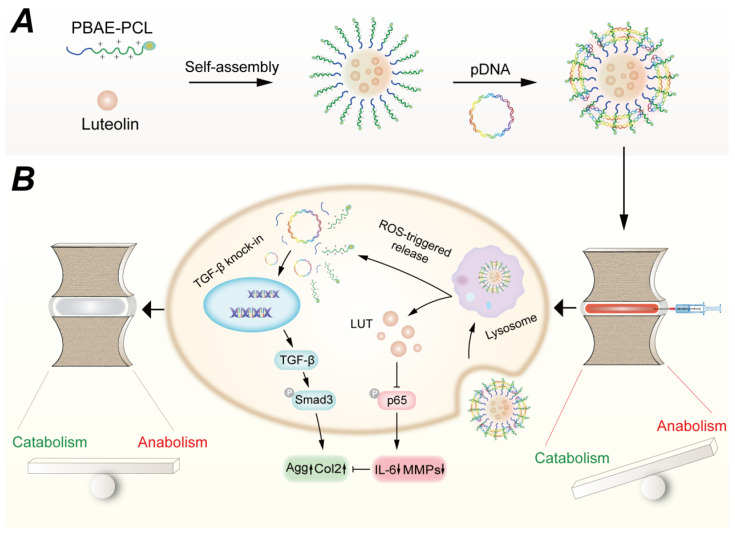
(**A**) ROS-responsive nanoplatform was developed for the combination therapy of LUT-TGF-β1 plasmid in IDD. (**B**) The molecular mechanisms underlying the therapeutic effect of the ROS-responsive nanoplatform in IDD. Reproduced with permission [16]. Copyright 2022, Tsinghua University Press.

The main biological function of heme oxygenase-1 is to convert harmful heme into antioxidants, suppress inflammation, and reduce oxidative stress. Feng et al. [47] used a composite cationic block copolymer gene delivery system, which combined two cationic polymer materials—polyethylene glycol-b-polyaspartic acid and poly(N-isopropylacrylamide)-b-polyaspartic acid—and co-incubated them with heme oxygenase-1 plasmids for gene delivery into NPCs. This composite was resistant to nucleases and had a strong resistance to protein adsorption, capable of reducing IL-1β-induced MMP expression, inhibiting the inflammatory response, and promoting the production of Col2a1 and Agg. After administration into the intervertebral discs of Sprague Dawley (SD) rats, it significantly suppressed the needle puncture-induced inflammatory response. Our group [16] employed polymeric nanoparticles based on poly(β-amino ester) to deliver luteolin and TGF-β plasmids for the co-delivery of drugs and genes to degenerated IVD (Figure 4A). This system not only suppressed inflammation but also promoted ECM production in NPCs, achieving ROS-responsive drug release within the disc and showing significant potential for promoting intervertebral disc regeneration (Figure 4B).

#### 3.3.2. Anti-Fibrosis

During IDD, NPCs undergo functional failure, leading to abnormal ECM metabolism, characterized by reduced synthesis of type II collagen, increased synthesis of type I collagen, and a decrease in the ratio of type II to type I collagen. This results in fibrosis of the nucleus pulposus and reduced cell function [4,48]. The loss of activity of the orphan nuclear receptor NR4A1 leads to sustained activation of the transforming growth factor-β (TGF-β) signaling pathway and pathological tissue fibrosis, making NR4A1 a potential new target for fibrosis therapy [49]. Feng et al. [50] synthesized a hyperbranched polymer carrier using polyethylene glycol (PEG) and low-molecular-weight polyether imide, which was complexed with anti-fibrosis NR4A1 plasmids and encapsulated in PLGA nanoparticles for controlled release. This system was then combined with nanofiber sponge microspheres to create a novel injectable scaffold for efficient plasmid transfection. The system achieved over 30 days of sustained release at the injection site in the rat tail, significantly inhibiting the synthesis of type I collagen and the ECM protein α-SMA, and suppressing fibrosis in the IVD of rats. This approach provided a new strategy for intervertebral disc regeneration and repair. Feng et al. [51] further designed a polymer micelle-based injectable hydrogel to deliver miR-29, which targets the Wnt/β-catenin pathway by inhibiting MMP2 expression. This approach reduces the expression of α-SMA and stress fibers, inhibiting fibrosis and promoting regeneration of the NP tissue.

#### 3.3.3. Promotion of Stem Cell Migration and Differentiation

Feng et al. [52] used nanofiber sponge microspheres developed by themselves as an injectable scaffold to load rabbit MSCs and anti-miR-199a. This system inhibited calcification and promoted MSC proliferation and differentiation. The nanofiber sponge microspheres scaffold, as an injectable platform, demonstrated significant potential for gene delivery applications in IDD. Table 1 summarizes the specific components and applications of the aforementioned gene-nanomaterial composites.

**Table 1:** Gene-nanoparticle complexes.

Type	Composition	Gene	Effect	References
Thermo-responsive mixed polyplex micelles	Poly(ethylene glycol)-block-poly [PEG-b-PAsp(DET)] and poly(*N*-isopropylacrylamide)-block-PAsp(DET) [PNIPAM-b-PAsp(DET)]	Heme oxygenase-1 (HO-1)	Delivery vector: resistance to nuclease decomposition and protein adsorption Gene: anti-inflammation	[47]
Nanofibrous spongy microspheres (NF-SMS) loaded hyperbranched polymer (HP)/PLGA (HP/NS)	Hyperbranched polymer (HP), PLGA, nanofibrous spongy microspheres (NF-SMS)	Orphan nuclear receptor-4A1 NR4A1	Delivery vector: sustained release over 30 days Gene: anti-fibrosis	[49]
Polyplex Micelle-Loaded Injectable Hydrogels	PEG_114_-GPLGVRG-PAsp(Det)_48_-Chole	miR-29	Delivery vector: MMP2 responsiveness Gene: anti-fibrosis	[51]
Nanofibrous spongy microspheres (NF-SMS) loaded hyperbranched polymer (HP)/PLGA (HP/NS)	Hyperbranched polymer (HP), PLGA, nanofibrous spongy microspheres (NF-SMS); MSCs	anti-miR-199a	Delivery vector: MSCs loading ability and promote their differentiation Gene: anti-calcification	[52]
ROS-responsive cationic copolymer co-delivery nanoparticles	Luteolin-pTGF-β1 plasmid@PBC(PBAE-PCL)	TGF-β1	Delivery vector: ROS-Responsiveness; Co-delivery Gene: promote ECM production	[16]

### 3.4. Fullerene Derivatives—Fullerol

Nanomaterials composed of carbon elements are collectively referred to as carbon nanomaterials. In 1963, Gott et al. discovered that carbon exhibits excellent anti-thrombotic properties during research on artificial blood vessels [53]. Subsequently, carbon materials have been widely applied in artificial blood vessels, artificial heart valves, artificial tooth roots, bones, joints, ligaments, tendons, and more. In 1985, the British scientist Kroto discovered fullerene (C60), and in 2004, Andre Geim and Konstantin Novoselov, from the University of Manchester, used transparent tape to prepare two-dimensional graphene materials. Carbon nanomaterials have since become a research hotspot in the field of materials science due to their unique structure and physicochemical properties. Currently, the carbon nanomaterials used in IDD are primarily fullerene derivatives—fullerol.

Fullerene is poorly soluble in water, which greatly limits its application in the biomedical field. Introducing hydroxyl groups onto fullerene can improve its water solubility. Fullerol is a common hydroxylated derivative of fullerene, which greatly expands its application field. It can suppress oxidative stress and inflammation by scavenging free radicals in the body [54]. The research group of Xudong Li at the University of Virginia has conducted in-depth studies on the application of fullerol in the treatment of IDD. The research team, including Liu et al. [55], isolated dorsal root ganglion cells from mice and induced inflammation with TNF-a. They found that fullerol nanoparticles could inhibit cell apoptosis, ROS levels, as well as the production of IL-6, COX-2, and PGE2, while also promoting the expression of antioxidant proteins (superoxide dismutase 2 and catalase) in dorsal root ganglion cells. In the same year, Liu et al. [56] also confirmed that fullerol nanoparticles could inhibit IL-1β-induced inflammation and adipogenic differentiation of vertebral bone marrow-derived MSCs. The inhibitory effect was dose-dependent, making fullerol nanoparticles a potential biotherapeutic approach for IDD. The research team of Yang et al. [57] used in vivo and in vitro rabbit IDD models to study the effect of fullerol nanoparticles in inhibiting IL-1β and H_2_O_2_-induced ECM degradation in NPCs. They confirmed that fullerol nanoparticles could delay IDD in rabbit experimental models.

### 3.5. Microspheres

Microspheres refer to microparticulate dispersion systems in which drugs are dispersed or adsorbed within polymer or macromolecular matrices. There are numerous materials for preparing microspheres, which are mainly categorized into natural polymer microspheres (e.g., gelatin microspheres, collagen microspheres) and synthetic polymer microspheres (e.g., poly(lactic-co-glycolic acid) microspheres, polyamide microspheres) [58]. In recent years, microsphere systems have garnered increasing attention in tissue engineering and have found expanding applications in IDD. The following review summarizes the applications of various microsphere systems in IDD, focusing on their anti-inflammatory properties and their ability to promote stem cell migration and differentiation.

#### 3.5.1. Anti-Inflammation

**Figure 5:**
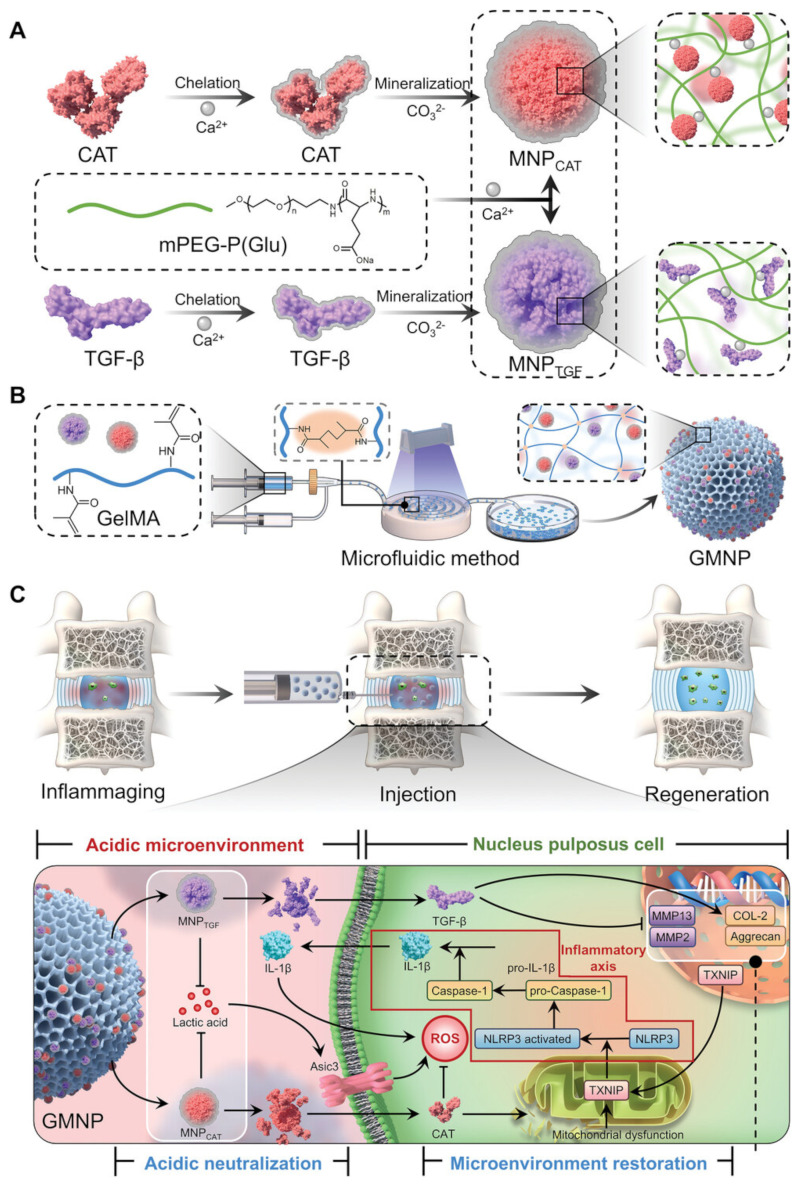
Schematic diagram of the preparation and therapeutic mechanism of hydrogen ion-absorbing hydrogel microspheres (GMNPs). (**A**) Formation of MNP incorporating CAT and TGF-β through biomimetic mineralization. (**B**) Fabrication of GMNP encapsulating MNP using a microfluidic approach. (**C**) The impact and mechanism of GMNP in stabilizing the microenvironment by absorbing hydrogen ions, inhibiting the NLRP3 inflammasome cascade, and promoting ECM synthesis. Reproduced with permission [63]. Copyright 2023, Wiley.

With the rapid advancement of synthetic polymer technology, absorbable and degradable polymers have evolved from aliphatic polymers to nitrogen-containing polymers, such as polyurethanes and polyamide esters, with enhanced biodegradability and controlled-release properties. Biodegradable polyamide ester-based microspheres represent a promising biomaterial for local drug delivery, as these amino acid-based polymers exhibit excellent thermal and mechanical properties [59]. This polymer can be degraded via enzymatic mechanisms, and the presence of numerous proteases in inflammatory environments, such as those in osteoarthritis and degenerated IVD, accelerates the degradation of the microspheres, enabling faster drug release and thereby controlling inflammation. Polyamide ester microspheres have been shown to possess excellent biocompatibility in L-929 fibroblasts as well as in rabbit and canine intervertebral discs, demonstrating anti-apoptotic effects at the cellular level, making them a promising safe, controlled-release system for IVD biotherapy [60]. Researchers have incorporated the cyclooxygenase-2 (COX-2) inhibitor celecoxib [61] and the glucocorticoid drug triamcinolone acetonide [62] into polyamide microspheres in a canine IDD model. Both drugs possess anti-inflammatory and antioxidant properties. The triamcinolone-loaded polyamide microspheres significantly reduce the expression levels of nerve growth factor, which contributes to pain, thereby alleviating pain associated with IDD. Celecoxib-loaded polyamide microspheres suppress prostaglandin E2 expression, and both in vitro and in vivo studies have confirmed their ability to halt the progression of IDD. Gorth et al. [30] loaded interleukin-1 receptor antagonist (IL-1Ra) into poly(lactic-co-glycolic acid) (PLGA) microspheres and analyzed the glycosaminoglycan content, nitric oxide production, and expression of inflammation-related genes following intervention in NPCs. The results demonstrated that IL-1Ra can inhibit IL-1β-induced inflammatory responses and restore the disc cell phenotype. Moreover, Cui et al. [63] designed a mineralized hydrogel microsphere (GMNP) with the ability to capture hydrogen ions using biomimetic mineralization and microfluidic techniques (Figure 5A,B). This GMNP system can neutralize accumulated lactic acid through the CaCO3 mineralized layer and eliminate excessive mitochondrial ROS, thereby inhibiting the NLRP3/Caspase-1/IL-1β cascade (Figure 5C). The GMNP system can enhance cell adhesion and reconstruct the ECM in vitro. The IDD rat model demonstrated that the GMNP system promotes radiographic improvement of IDD, facilitates NPCs regeneration, and suppresses the inflammatory process.

#### 3.5.2. Promotion of Stem Cell Migration and Differentiation

Gelatin is a biodegradable and biocompatible biomaterial, widely used in the pharmaceutical field and the drug industry. It can load bioactive factors and cells on its surface, enabling sustained drug release. Gelatin nanoparticles also provide a growth microenvironment for living organisms, promoting stem cell migration and differentiation [64]. Xia et al. [65] prepared gelatin microspheres that sustainably release growth differentiation factor-5 (GDF-5) and used them as a delivery vehicle for nucleus pulposus-like cells. As gelatin degrades, GDF-5 is continuously released into the nucleus pulposus tissue to inhibit IDD and promote notochordal cell differentiation into nucleus pulposus-like cells. Furthermore, gelatin microspheres provide a carrier microenvironment for nucleus pulposus-like cells, aiding their survival in the degenerative inflammatory IVD microenvironment. When nucleus pulposus-like cells-GDF-5-loaded gelatin microspheres were injected into rat tail discs, IVD height and water content were measured using X-ray imaging and magnetic resonance imaging. Hematoxylin and eosin, Safranin O-fast green, and immunohistochemical staining results showed that nucleus pulposus-like cells-GDF-5-loaded gelatin microspheres could restore IVD height and water content. Tsaryk et al. [66] prepared collagen low-molecular-weight hyaluronic acid semi-interpenetrating network gelatin microspheres, which promote the growth of MSCs and nasal chondrocytes in vitro and in vivo and their differentiation into chondrocytes.

More than 70% of the organic material in bones is collagen, and in NP tissue, aside from water, the main components are type II and type I collagen. Collagen microsphere systems can provide a growth microenvironment for IVD tissue regeneration, promote stem cell migration and differentiation into NPCs, and facilitate functional reconstruction, guiding deposition in regions rich in glucosamine. Yuan et al. [67] designed a 3D collagen microsphere culture system, which better preserved the phenotypic characteristics of NPCs and promoted the synthesis of collagen matrix compared to traditional monolayer culture. Li et al. [68] designed collagen microcapsules that encapsulated MSCs within solid-phase microspheres made from a collagen nanofiber network. These solid-phase porous microspheres can support MSCs attachment, survival, proliferation, migration, differentiation, and matrix remodeling. In comparison with MSCs directly suspended in physiological saline for the treatment of rabbit degenerated IVD, after 6 months of administration, histological, biochemical, and biomechanical evaluations of the discs showed that MSCs in collagen microspheres performed better in maintaining dynamic mechanical behavior than MSCs in saline. Furthermore, based on macroscopic appearance, imaging, and histological examination of the IVD, the MSC–collagen microsphere delivery system significantly reduced the risk of osteophyte formation compared to saline. Additionally, synthetic polymer microspheres—poly(lactic-co-glycolic acid) (PLGA) microspheres—loaded with dexamethasone and basic fibroblast growth factor can also serve as scaffold materials to promote the growth of rat MSCs and their differentiation into NPCs, while alleviating inflammation in IVD [69].

### 3.6. Biological Source Nanocarriers—Exosomes

Exosomes are nanoscale (40–120 nm) vesicles with a bilayer membrane structure secreted by cells through budding. They are rich in cell-derived mRNA, miRNA, and protein components, and these components can enter target cells to exert corresponding biological functions, playing a protective role in damage to the heart, kidneys, blood vessels, and nerves [70]. MSC-derived exosomes are a novel drug and gene delivery system [71]. Additionally, there are reports indicating that exosomes derived from NPCs can intervene in IDD by inhibiting NPCs apoptosis, oxidative stress, and fibrosis, promoting disc repair. The primary mechanism of action is through miRNA and related signaling pathways. Exosomes from different cell sources have been widely used in IVD repair and regeneration.

#### 3.6.1. Exosomes Derived from Bone Marrow Mesenchymal Stem Cells (BMSCs)

Zhu et al. [72] isolated exosomes from BMSCs and confirmed that miR-142-3p can alleviate apoptosis and inflammation in NPCs by targeting MLK3 and further regulating the activation of the MAPK pathway. Another miRNA secreted by BMSCs-derived exosomes, miR-532-5p, may inhibit TNF-a-induced nucleus pulposus cell apoptosis, ECM degradation, and fibrosis deposition by targeting the RASSF5 gene [73]. In animal studies, injection of MSCs-derived exosomes into the IVD of SD rats can inhibit disc cell apoptosis and degeneration. miR-21 plays a key role in blocking nucleus pulposus cell apoptosis and alleviating the loss of cell phenotype. The mechanism may involve inhibition of the PTEN/PI3K/Akt pathway, thereby reducing apoptosis in NPCs. In addition to inhibiting apoptosis in NPCs, MSCs-derived exosomes can also repair intervertebral disc regeneration by modulating endoplasmic reticulum stress in NPCs through the AKT/ERK pathway [74]. Interestingly, a study [75] showed that a protein derived from MSCs exosomes—mitochondrial-associated protein—can target the NLRP3 inflammasome. By repairing damaged mitochondria, it inhibits H_2_O_2_-induced inflammation and reactive oxygen species production in NPCs. It was also confirmed in a New Zealand White rabbit model that this protein prevents the progression of IVD degenerative disease. Pyroptosis, also known as inflammatory necrosis, plays a crucial role in the occurrence and development of various inflammatory diseases. Zhang et al. [76] found that NLRP3-mediated pyroptosis was activated in the IDD mouse model and in LPS-induced NPCs. MSCs-derived exosomes inhibit pyroptosis by suppressing the NLRP3 pathway, thereby preventing IDD. This effect may be associated with miR-410 derived from MSCs exosomes, which can directly bind to NLRP3 mRNA. Su et al. [77] analyzed miRNA profiles in BMSCs and identified miR-145a-5p as the primary miRNA carried by BMSCs. Overexpression of miR-145a-5p can inhibit the catabolism of NPCs through the miR-145a-5p/USP31/HIF-1α signaling pathway.

#### 3.6.2. Exosomes Derived from NPCs

Lan et al. [78] found that exosomes derived from human NPCs can induce MSCs to differentiate into nucleus pulposus-like cells in vitro. Rab27a-siRNA can block this process, suggesting the important role of Rab27a in exosomes, and inhibiting the Notch1 pathway can promote differentiation. Moen et al. [79] revealed that miRNA-223 in NPCs-derived exosomes can reduce the production of IL-6 and nitric oxide, inhibit harmful spinal cord signaling, and alleviate nerve root pain in SD rats. In recent years, some circular RNAs (circRNAs) have been confirmed to be enriched with miRNA binding sites and interact with miRNAs as endogenous competitive RNAs, affecting the expression of target mRNAs and ultimately regulating pathophysiological processes [80]. Exosomes derived from NPCs carry circRNA_0000253, which promotes IDD by adsorbing miRNA-141-5p and downregulating SIRT1. CircRNA_0000253 may be a potential target for treating IDD.

#### 3.6.3. Exosomes Derived from Human Placenta/Umbilical Cord-Derived Mesenchymal Stem Cells (PLMSCs/ULMSCs)

Compared to other stem cells, PLMSCs/ULMSCs are easy to source, abundant in quantity, and easy to isolate, culture, expand, and purify, making exosomes derived from them more accessible. Exosomes derived from PLMSCs deliver AntagomiR-4450 (EXO-AntagomiR-4450) to target zinc finger protein 121 (ZNF121) [81], thereby inhibiting TNF-a-induced inflammation and apoptosis in NPCs. Animal experiments show that these exosomes can effectively improve gait abnormalities in rats. PLMSCs-derived exosomes may be a viable nanocarrier for delivering inhibitory oligonucleotides. In a recent study, Shu et al. [82] investigated the therapeutic potential of ULMSCs in treating IDD by reducing mitochondrial reactive oxygen species (ROS) and improving mitochondrial function. The results demonstrated that ULMSCs effectively restored ECM and alleviated mitochondrial oxidative stress in NPCs both in vitro and in vivo, suggesting their potential as a treatment for IDD. Table 2 delineates the exosomes derived from diverse sources, elucidating their respective mechanisms of action.

### 3.7. Nanocomposite Hydrogels Improve the Mechanical Properties of the IVD

With the in-depth study of the pathophysiological changes in IDD, the application of hydrogel-based biomaterials has gradually expanded. Traditional chemically crosslinked hydrogels have poor mechanical properties, which limits their use in many fields. Nanocomposite hydrogels, due to their simple preparation methods and excellent mechanical properties, have attracted widespread attention from researchers [83]. Nanocomposite hydrogels involve the incorporation of nanoparticles or nanostructures into the molecular network of the hydrogel through physical or covalent crosslinking methods. Due to the unique physicochemical properties of nanoparticles, hydrogels that incorporate nanomaterials can enhance their mechanical properties, improve biocompatibility, and promote stem cell migration and differentiation. Nanocomposite hydrogels applied in IDD mainly include composite hydrogels made from synthetic materials (such as polyethylene glycol, graphene), natural materials (such as cellulose), and natural–synthetic material composite hydrogels (e.g., N-acetylglucosamine-silk fibroin; PEG dimethacrylate-cellulose; chitosan-polyhydroxybutyrate-co-valerate-chondroitin sulfate). Synthetic materials offer strong controllability, enabling the addition of various modifications, and exhibit good mechanical properties, though their biocompatibility is somewhat poor [84]. Natural materials have excellent biocompatibility and biodegradability [85]. Natural–synthetic materials combine the advantages of both, including superior mechanical properties and biocompatibility, making them a promising direction for future research in IVD regeneration.

#### 3.7.1. Synthetic Material-Reinforced Hydrogels

In the application of synthetic material-reinforced hydrogels, there has been extensive research on polyethylene glycol (PEG) nanogels. However, reports on the synthesis of PEG nanogels with high mechanical strength are rare. Nguyen et al. [86] synthesized a novel PEG nanocomposite hydrogel with pH-responsive properties and controllable mechanical strength through the ring-opening reaction of diglycerol ether and carboxylate ions. The compression fracture strain is greater than 98%, the fracture strain energy density is as high as 1.88 MJm-3, and the tensile fracture strain is 230%. This is one of the strongest PEG nanogels in terms of compression performance reported in the literature to date. It enables better resistance to torque deformation after implantation into the IVD, providing better cushioning ability for the IVD tissue. After co-culturing human NPCs with the system for 8 days, high cell viability remained. These results show the potential of PEG nanogels for IVD repair and regeneration. Another type of hydrogel composite with synthetic materials is the graphene oxide self-assembled peptide hydrogel [87], which enhances the mechanical strength of the hydrogel system by adding graphene oxide. Through in vitro 3D NPCs culture, it was found that graphene oxide can maintain the metabolic activity of cells and has good biocompatibility.

#### 3.7.2. Natural–Synthetic Composite Hydrogels

In the application of natural–synthetic composite hydrogels in intervertebral disc degeneration, the primary natural materials used in the composite hydrogels include cellulose, silk fibroin, and chondroitin sulfate. Pereira et al. [88] synthesized a gellan gum loaded with nanocrystalline cellulose, which enhanced the mechanical stiffness of the system. The compression mechanical tests showed that the compressive modulus was close to that of human AF tissue, and high cell viability was maintained after 14 days of co-culture with bovine AF cells. Schmocker et al. [89] reported a light-polymerized PEG dimethacrylate nanocellulose composite hydrogel, where the introduction of nanocellulose enhanced the structural strength of the hydrogel. Due to its excellent biocompatibility, the composite hydrogel did not significantly affect the water saturation, and when implanted into bovine IVD, it significantly restored disc height. After 500,000 fatigue cycles, the disc height remained unchanged. Murab et al. [90] encapsulated N-acetylglucosamine in silk fibroin hydrogel. The assembled silk fibroin hydrogel provided sufficient structural support for ex vivo degenerated IVD in cyclic compression tests, similar to those in natural IVD. The controlled release of N-acetylglucosamine from silk fibroin hollow microspheres enhanced the proteoglycan production in human adipose-derived stem cells, indicating the potential of injectable silk fibroin microspheres–silk fibroin hydrogel for regenerating degenerated disc tissue. Nair et al. [91] prepared chitosan-polyhydroxybutyrate-co-pentanoic acid and chondroitin sulfate nanocomposite hydrogels, which could withstand different stresses corresponding to daily activities such as lying down (0.01 MPa), sitting (0.5 MPa), and standing (1.0 MPa) under dynamic conditions, showing stable hardness, elastic modulus, and viscous modulus. Chitosan nanoparticles significantly enhanced the activity of MSCs and their ability to differentiate into chondrocytes.

## 4. Conclusions and Future Perspectives

Nanomedicine plays an important role in IVD repair and regeneration, providing new approaches for the treatment of IDD. Despite significant progress in the use of related nanomaterials for tissue engineering of IDD, it remains at the basic experimental level and there is still a distance to clinical translation. Currently, no nanotherapy has been approved by the FDA for clinical treatment of spinal diseases. The reasons for the limitations in its application development are multifaceted. First, do the animal models of IDD accurately reflect the pathophysiological processes of clinical IDD? IDD is a process that occurs with aging, involving a decrease in water content in the AF and NP, as well as changes in biochemical components such as proteins. The NP loses elasticity, and the AF gradually develops fissures. The most widely used method for creating IDD animal models in basic scientific research is the needle puncture method [92,93]. This involves direct puncture of the animal’s disc using a syringe needle of a specific diameter, causing tear in the AF and loss of NP tissue, thereby simulating clinical IDD. This method is simple to perform and has good reproducibility. However, the puncture method, which only involves a single factor, leads to an acute degeneration process, which does not align with the multifactorial, chronic pathophysiological changes involved in clinical IDD. Developing animal models that more closely mimic the true process of disc degeneration will be a key direction for future research.

Secondly, the structure of the IVD is unique, with no blood supply. Systemic drug administration makes it difficult for the drug to accumulate, limiting its therapeutic effect. Although one study [33] has developed a method for systemic drug delivery, its biological safety and efficacy in humans are still debatable. The IVD is not located on the surface of the body. Its dorsal side is covered by multiple layers, including ligaments, muscles, and spinous processes. In experimental studies, traditional local drug delivery methods must pass through these structures and the AF surrounding the NP. Drug administration itself causes damage to the IVD, which may accelerate degeneration. Therefore, the development of new drug delivery methods that can target the IVD and improve drug accumulation is an urgent research priority.

Nanomedicine holds promise in helping researchers address the issue of targeted accumulation in future studies. During the process of clinical translation, the following issues should be considered: 1. Improve the stability of the nanomaterial system, especially for drugs delivered through intravenous systemic administration. Stability determines the biological safety of the nanomaterial system, and is the first step toward clinical translation. 2. Increase the drug loading capacity, which is very important for the treatment of IDD. Each in situ injection into the disc increases damage to the skin and surrounding soft tissues. Increasing the drug loading capacity means reducing the number of injections and thereby minimizing toxicity to normal organs and tissues. 3. Develop new targeted drug delivery nanomaterials with better biocompatibility, biodegradability, sustained release and targeting delivery capabilities.

Gene therapy has made a series of research achievements in the field of cancer diseases, and research in other disease areas is also in full swing. The study of non-viral nanomaterial-based gene delivery systems is in its infancy in the field of IDD, and has higher safety compared to viral vectors. However, we still need to develop new nanodelivery materials with better biocompatibility and controlled release capabilities, aiming to move from basic research to clinical applications. At the same time, we should also seek more suitable target genes, or even multiple genes, to provide controllable interventions for IDD. A single gene alteration often cannot effectively control the progression of IDD, especially in patients with moderate to severe IDD. Future gene therapy research may need to focus on multi-gene interventions at different times and spaces to manage IDD.

Additionally, exosomes, as nano-sized biological vesicles, can deliver specific genes and drugs for the intervention of IDD. Several studies have demonstrated the tremendous potential of exosomes in the treatment of IDD, but they are still in the early stages of development and face some issues. Exosome heterogeneity and low production efficiency are the main obstacles to their application, and optimizing production methods to improve yield is urgently needed. The proteins and genes in exosomes are complex, and the mechanisms and precise contents involved need further study to improve the homogeneity of exosome extraction.

Among the diverse nanomaterials discussed in this review, nanoparticles exhibit superior drug-loading capacity and tunable surface properties; however, they encounter significant challenges in achieving targeted delivery via non-local administration and penetrating the dense ECM of the IVD. Gene-nanocomplexes enable genetic material delivery for regenerative therapy but suffer from relatively low transfection efficiency. Microspheres provide sustained drug release but lack mechanical properties and may cause inflammation by their degradation products, such as lactic acid from PLGA. Exosomes, as natural nanocarriers, mediate cell-to-cell communication but are difficult to produce at scale. Among these, nanocomposite hydrogels stand out as the most promising due to their ability to closely mimic the mechanical environment of the intervertebral disc, providing essential support for regeneration. By incorporating functionalized nanoparticles, nanocomposite hydrogels can achieve multi-functional properties, such as controlled drug release, anti-inflammatory effects, and enhanced cell adhesion, enabling comprehensive and multi-level IVD regeneration. Although challenges remain in optimizing degradation rates and long-term stability, their unique combination of mechanical and bioactive properties makes them a highly prospective platform for IDD therapy.

Overall, while nanomedicine and nanomaterials show great potential in the treatment of IDD, there are still many challenges, particularly in animal models, drug delivery methods, and clinical translation. Future research should focus on optimizing existing technologies, enhancing the biocompatibility, biodegradability, and targeted delivery capabilities of nanomaterials, to provide more effective solutions for the clinical treatment of IDD.

## Figures and Tables

**Table 2:** Exosomes.

Source of Exosome	Therapeutic Agent	Signal Pathway	Effect	Tests	Animal Model	Reference
Bone marrow-derived MSCs (BMSCs)	miR-142-3P	MLK3/MAPK	Inhibit NPs apoptosis	In vitro	-	[72]
BMSCs	miR-532-5p	RASSF5	Inhibit NPs apoptosis and fibrosis	In vitro	-	[73]
BMSCs	miR-21	PTEN/PI3K/Akt	Inhibit NPs apoptosis	In vitro/in vivo	SD Rat	[74]
BMSCs	Mitochondria-related proteins	TXNIP/NLRP3	Inhibit NPs inflammation and oxidative stress	In vitro/in vivo	Rabbit	[75]
BMSCs	miR-410	NLRP3	Inhibit NPs pyroptosis	In vitro/in vivo	C57BL/6	[76]
Human placental MSCs (PLMSCs)	AntagomiR-4450	miR-4450/ZNF121	Inhibit NPs inflammation and apoptosis	In vitro/in vivo	SD Rat	[81]
Human umbilical cord MSCs (ULMSCs)	-	miR-194-5p/TFAM	Inhibit mitochondrial oxidative stress	In vitro/in vivo	SD Rat	[82]
NPCs	Rab27a	Notch1	Promote differentiation of MSCs into NPs	In vitro	-	[78]
NPCs	miR-223	-	Inhibit lumbar neuronal pain	In vitro/in vivo	SD Rat/Human	[79]
NPCs	circRNA_0000253	miRNA-141-5p/SIRT1	Promote IDD	In vitro/in vivo	SD rat	[80]
